# Role of endothelial cells in tumor microenvironment

**DOI:** 10.1002/ctm2.450

**Published:** 2021-06-20

**Authors:** Dawei Yang, Peipei Guo, Tianrui He, Charles A. Powell

**Affiliations:** ^1^ Department of Pulmonary and Critical Care Medicine Zhongshan Hospital Institute for Clinical Science, Shanghai Medical College Shanghai Engineering Research Center of AI Technology for Cardiopulmonary Diseases Shanghai Engineer & Technology Research Center of Internet of Things for Respiratory Medicine Zhongshan Hospital Fudan University Shanghai 200032 China; ^2^ Division of Pulmonary, Critical Care and Sleep Medicine Icahn School of Medicine at Mount Sinai New York New York USA; ^3^ Zai Lab Shanghai China

To the Editor:

Endothelial cells line the vascular system and play essential roles in regulating tumor initiation, progression, and metastasis. The application of single‐cell RNA sequencing has identified distinct lineages of endothelial cells during the spatial–temporal evolution of the tumor microenvironment (TME). Subgroups of endothelial cells exist that either promote or prevent tumor progression from non‐invasive to the invasive stage. Mechanisms of functional tumor endothelial cells (TECs) include cytokine secretion, which activates receptors on the tumor cells and/or suppresses antitumor immune reaction via attenuating the cytotoxic responses of the immune cells (Figure 1). There are currently few available methods to detect TECs in the clinical setting; it would be a promising approach to leverage TECs targeted therapy to improve the current treatment regimens for early‐stage cancer.

**FIGURE 1:**
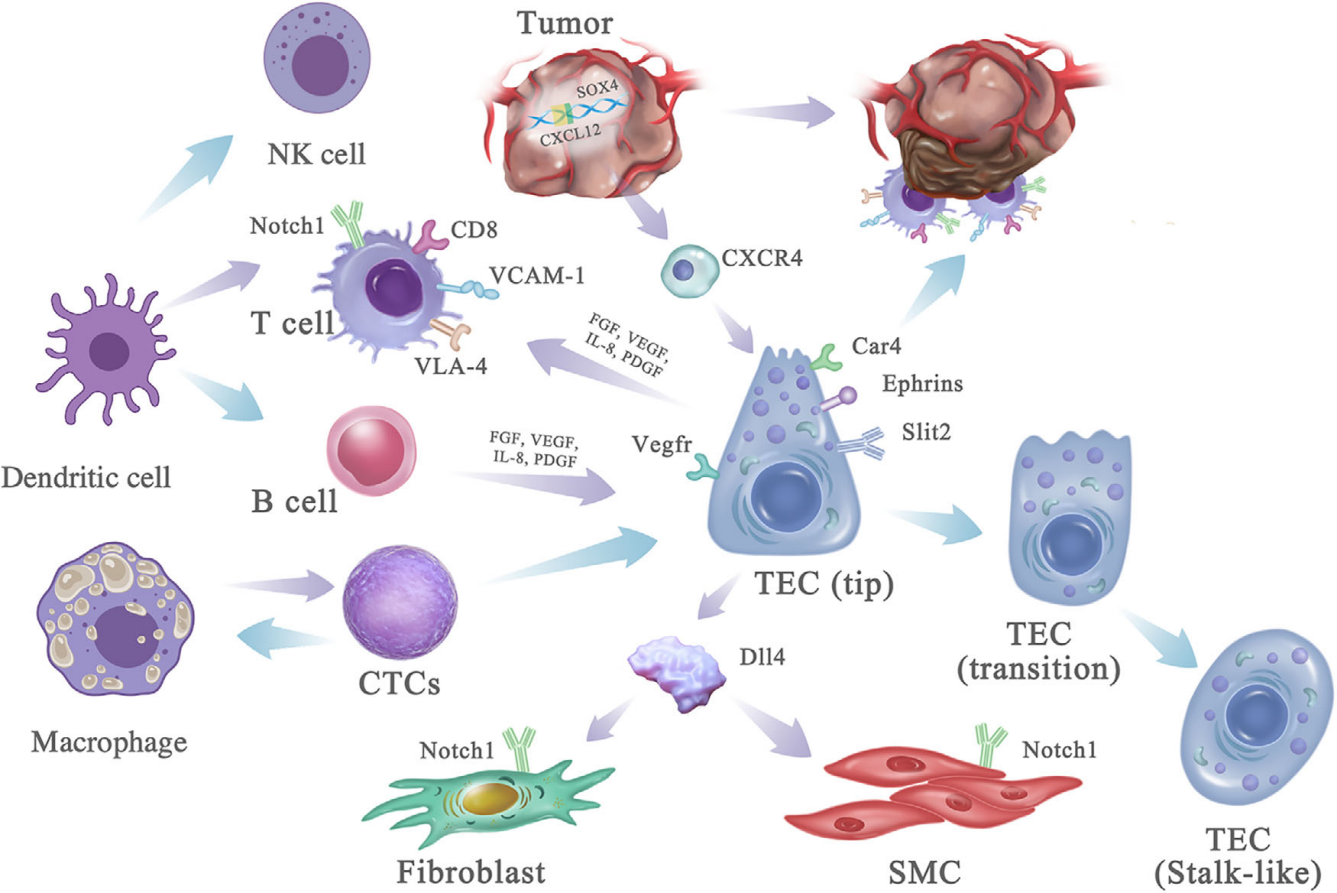
Schematic of tumor microenvironment in the early stage of the malignant tumor

**FIGURE 2:**
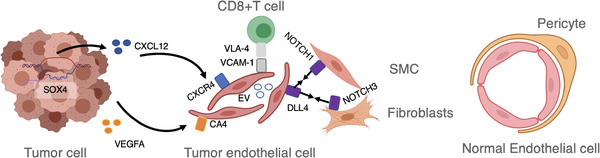
Illustration of gene‐ and regulation‐based molecular mechanisms in tumor endothelial microenvironment

TECs could promote cancer progression by supporting tumor metabolism^1^ or secreting paracrine factors.^2^ Recently, by single‐cell RNA sequencing (scRNA‐seq), we could gain a landscape view of the dynamic gene network in the TECs.^3^ For example, in a metastatic melanoma study, Tirosh et al. identified that the TECs in the drug‐resistant tumors harbored heterogeneous genetic signal compared to the normal ECs gene expression.^4^ The scRNA‐seq analysis aims to discover the unique biomarker or signaling ligands/receptors on the cell population of interest. For example, one scRNA‐seq profiling of mouse lung endothelial cells defined a specific lung endothelial cell population,^5^ marked by carbonic anhydrase 4 (CA4), whose expression depends on VEGFA expression AT1‐positive cells.^6^ The Notch ligand Dll4 was abundantly expressed by endothelial cells, impacting smooth muscle cells (SMC) and fibroblasts by binding with their Notch1 or Notch3 receptors (Figure 2).^7^ The other endothelial‐specific markers include Ephrins^8^ and Slit2,^9^ as well as TEC specific tip cell‐specific marker CXCR4, PGF and LXN, etc.^10^

The VEGF and Notch signaling pathways in tumor ECs could be more active than in the normal tissue endothelial cells, responsible for the upregulation of angiogenesis.^11^ The downregulation of gene expression related to immune activation in tumor ECs indicates that tumor ECs would suppress the antitumor immune function. VEGF or Dll4‐Notch signaling upregulations to play decisive roles in tumor formation and metastasis, probably through VEGF or Dll4‐mediated formation of TEC subgroups (i.e., tip‐like, transition, and stalk‐like cells) (Figure 1), as evidenced by scRNA‐seq profiles and tip TEC signature genes are well related with patient survival.^10^

scRNA‐seq studies can also reveal the impact of tumor cells on TECs. The aberrant gene within tumors could activate the *CXCL12* promoter within tumors, which could bind and activate CXCR4 signaling in endothelial cells, induce neovascularization, and promote distant metastasis in hepatocellular carcinoma cells.^12^, ^13^ The tumor‐initiating cells could impact surrounding endothelial cells by transferring oncogenic sequences to TECs via extracellular vehicles (EVs), which subsequently induces micronuclei formation, along with TEC migration and proliferation (Figure 2).^14^, ^15^

Thus, the identification and validation of TEC‐specific markers will improve the current diagnosis for early detection of TMEs. Wang et al. developed an apelin‐based synNotch receptors binding system to detect the angiogenic signaling within tumor.^16^ Based on scRNA‐seq, one subgroup of TEC, tip TECs, correlated with patient survival. Therefore, Tip TECs can be considered a potential marker to evaluate the effectiveness of VEGF blockade therapies.^10^ Besides, a higher proportion of endothelial cells can be harvested in clinical core biopsy samples than in surgical resection samples.^17^ Due to the technical difficulties of obtaining TECs, one approach is to apply a set of transgenetic reporter mice to capture endothelial cells in mouse models. He et al. used this method to apply scRNA‐seq on mouse brain and lung endothelial cells and studied the vascular populations and transcriptome during development and disease.^18^

In addition to the interaction between tumor cells and TECs, other types of cells in TME (e.g., tumor‐associated macrophage and cancer‐associated fibroblasts) also engage in cell–cell communications with TECs.^19^ Dendritic cells were one of the most abundant cells in the immune microenvironment,^20^ glioma‐associated macrophages have an emerging role in the promotion of tumor proliferation, invasion, and metastasis and also stimulation of neoangiogenesis,^21^, ^22^ the intense interrelation between immune cells and extracellular matrix molecules in building the TME,^23^, ^24^ NK cells are also one important subset of tumor‐antagonizing immune cells that mediate the immunosurveillance of tumor.^25^ The induction of retinoblastoma protein (Rb)‐mediated senescence on KRAS mutant pancreatic ductal adenocarcinoma could produce pro‐angiogenic factors and promote the tumor vasculature normalization, which in turn enhance drug delivery and efficacy of cytotoxic gemcitabine chemotherapy.^26^, ^27^ Meanwhile, endothelial cell activation could contribute to CD8^+^ T cells' infiltration by induction of VCAM‐1, which binds with VLA‐4 on CD8^+^ T cells. Similarly, IgG4^+^CD49b^+^CD73^+^ B cells expressing pro‐angiogenic cytokine could efficiently promote endothelial cell tubes' formation in the tumor microenvironment.^28^

Regarding translational therapy, historically, anti‐angiogenesis therapy targets tumor vascular network growth, which mainly consists of two mechanisms: (a) angiogenesis, the formation of new blood vessels from existing vessels, and (b) vasculogenesis, the de novo formation of blood vessels from endothelial precursors.^29^ Many growth factors promote angiogenic output (e.g., FGF, VEGF, IL‐8, and PDGF) and endogenous inhibitors also exist to block vessel growth (e.g., thrombospondin, tumstatin, canstatin, endostatin, angiostatin, and interferon‐alpha/beta).^30^ However, the targeted inhibition of the VEGF signaling has not always resulted in favorable outcomes in cancer patients' treatment.^31^ Recently, the combination of anti‐PD‐L1 and anti‐VEGF therapy showed significant benefit in patients with unresectable hepatocellular carcinoma.^32^ It is hinted that the anti‐VEGF neutralizing antibody reversed the immunosuppressive function of TECs and promoted T‐cell infiltration in the tumor. This warrants a better understanding of the complex interactions between tumor and TECs.

Also, scRNA‐seq of TECs provides the potential to identify more targeted therapy based on the cellular and molecular changes within TECs, and overcome the limitations of anti‐angiogenesis therapies. Furthermore, a subgroup of circulating tumor cells (CTCs) population in colorectal cancer patients' blood was TECs^33^; the screening of TECs in patients' peripheral blood with different stages of tumor has the potential to serve as a biomarker for early tumor diagnosis. It would be valuable to define the critical regulatory notes within the TEC gene expression network, explore their expression by multiomics, and, most importantly, to integrate regulatory nodes' expression with patients' clinical phenomes by way of clinical transomics.^34^, ^35^ With the rapid development of biotechnologies, a deep understanding of the crosstalk between tumor cells and TECs at the early stage of tumorigenesis will help discover more precise and potent biomarkers and improve clinical outcomes for cancer treatment.

## References

[1] Yeh WL , Lin CJ , Fu WM . Enhancement of glucose transporter expression of brain endothelial cells by vascular endothelial growth factor derived from glioma exposed to hypoxia. Mol Pharmacol. 2008;73:170‐177. 17942749 10.1124/mol.107.038851

[2] Cao Z , Ding B‐S , Guo P , et al. Angiocrine factors deployed by tumor vascular niche induce B cell lymphoma invasiveness and chemoresistance. Cancer Cell. 2014;25:350‐365. 24651014 10.1016/j.ccr.2014.02.005 PMC4017921

[3] Kalucka J , de Rooij LPMH , Goveia J , et al. Single‐cell transcriptome atlas of murine endothelial cells. Cell. 2020;180:764‐779.e20. 32059779 10.1016/j.cell.2020.01.015

[4] Tirosh I , Izar B , Prakadan SM , et al. Dissecting the multicellular ecosystem of metastatic melanoma by single‐cell RNA‐seq. Science. 2016;352:189‐196. 27124452 10.1126/science.aad0501 PMC4944528

[5] Gillich A , Zhang F , Farmer CG , et al. Capillary cell‐type specialization in the alveolus. Nature. 2020;586:785‐789. 33057196 10.1038/s41586-020-2822-7 PMC7721049

[6] Vila Ellis L , Cain MP , Hutchison V , et al. Epithelial VEGFA specifies a distinct endothelial population in the mouse lung. Dev Cell. 2020;52:617‐630.e6. 32059772 10.1016/j.devcel.2020.01.009 PMC7170573

[7] Raredon MSB , Adams TS , Suhail Y , et al. Single‐cell connectomic analysis of adult mammalian lungs. Sci Adv. 2019;5:eaaw3851. 31840053 10.1126/sciadv.aaw3851 PMC6892628

[8] Pandey A , Shao H , Marks RM , Polverini PJ , Dixit VM . Role of B61, the ligand for the Eck receptor tyrosine kinase, in TNF‐alpha‐induced angiogenesis. Science. 1995;268:567‐569. 7536959 10.1126/science.7536959

[9] Rama N , Dubrac A , Mathivet T , et al. Slit2 signaling through Robo1 and Robo2 is required for retinal neovascularization. Nat Med. 2015;21:483‐491. 25894826 10.1038/nm.3849 PMC4819398

[10] Goveia J , Rohlenova K , Taverna F , et al. An integrated gene expression landscape profiling approach to identify lung tumor endothelial cell heterogeneity and angiogenic candidates. Cancer Cell. 2020;37:21‐36.e13. 31935371 10.1016/j.ccell.2019.12.001

[11] Kim N , Kim HK , Lee K , et al. Single‐cell RNA sequencing demonstrates the molecular and cellular reprogramming of metastatic lung adenocarcinoma. Nat Commun. 2020;11:2285. 32385277 10.1038/s41467-020-16164-1 PMC7210975

[12] Tsai C‐N , Yu S‐C , Lee C‐W , et al. SOX4 activates CXCL12 in hepatocellular carcinoma cells to modulate endothelial cell migration and angiogenesis in vivo. Oncogene. 2020;39(24):4695‐4710. 32404985 10.1038/s41388-020-1319-z

[13] Williamson T , Sultanpuram N , Sendi H . The role of liver microenvironment in hepatic metastasis. Clin Transl Med. 2019;8:21. 31263976 10.1186/s40169-019-0237-6 PMC6603103

[14] Chennakrishnaiah S , Tsering T , Gregory C , et al. Extracellular vesicles from genetically unstable, oncogene‐driven cancer cells trigger micronuclei formation in endothelial cells. Sci Rep. 2020;10:8532. 32444772 10.1038/s41598-020-65640-7 PMC7244541

[15] Shah TG , Predescu D , Predescu S . Mesenchymal stem cells‐derived extracellular vesicles in acute respiratory distress syndrome: a review of current literature and potential future treatment options. Clin Transl Med. 2019;8:25. 31512000 10.1186/s40169-019-0242-9 PMC6739436

[16] Wang Z , Wang F , Zhong J , et al. Using apelin‐based synthetic Notch receptors to detect angiogenesis and treat solid tumors. Nat Commun. 2020;11:2163. 32358530 10.1038/s41467-020-15729-4 PMC7195494

[17] Slyper M , Porter A , Ashenberg O , et al. A single‐cell and single‐nucleus RNA‐Seq toolbox for fresh and frozen human tumors. Nat Med. 2020;26:792‐802. 32405060 10.1038/s41591-020-0844-1 PMC7220853

[18] He L , Vanlandewijck M , Mae MA , et al. Single‐cell RNA sequencing of mouse brain and lung vascular and vessel‐associated cell types. Sci Data. 2018;5:180160. 30129931 10.1038/sdata.2018.160 PMC6103262

[19] Zhang L , Ziyi L , Skrzypczynska KM , et al. Single‐cell analyses inform mechanisms of myeloid‐targeted therapies in colon cancer. Cell. 2020;181:442‐459.e29. 32302573 10.1016/j.cell.2020.03.048

[20] Deng X , Lin D , Zhang X , et al. Profiles of immune‐related genes and immune cell infiltration in the tumor microenvironment of diffuse lower‐grade gliomas. J Cell Physiol. 2020;235:7321‐7331. 32162312 10.1002/jcp.29633

[21] Hu F , Dzaye OD , Hahn A , et al. Glioma‐derived versican promotes tumor expansion via glioma‐associated microglial/macrophages Toll‐like receptor 2 signaling. Neuro Oncol. 2015;17:200‐210. 25452390 10.1093/neuonc/nou324 PMC4288527

[22] Zhu C , Kros JM , Cheng C , Mustafa D . The contribution of tumor‐associated macrophages in glioma neo‐angiogenesis and implications for anti‐angiogenic strategies. Neuro Oncol. 2017;19:1435‐1446. 28575312 10.1093/neuonc/nox081 PMC5737221

[23] Jia D , Li S , Li D , et al. Mining TCGA database for genes of prognostic value in glioblastoma microenvironment. Aging (Albany NY). 2018;10:592‐605. 29676997 10.18632/aging.101415 PMC5940130

[24] Yang S , Liu T , Nan H , et al. Comprehensive analysis of prognostic immune‐related genes in the tumor microenvironment of cutaneous melanoma. J Cell Physiol. 2020;235:1025‐1035. 31240705 10.1002/jcp.29018

[25] Lei X , Lei Y , Li J‐K , et al. Immune cells within the tumor microenvironment: biological functions and roles in cancer immunotherapy. Cancer Lett. 2020;470:126‐133. 31730903 10.1016/j.canlet.2019.11.009

[26] Ruscetti M , Morris JP 4th , Mezzadra R , et al. Senescence‐induced vascular remodeling creates therapeutic vulnerabilities in pancreas cancer. Cell. 2020;181:424‐441.e21. 32234521 10.1016/j.cell.2020.03.008 PMC7278897

[27] Daniel SK , Sullivan KM , Labadie KP , Pillarisetty VG . Hypoxia as a barrier to immunotherapy in pancreatic adenocarcinoma. Clin Transl Med. 2019;8:10. 30931508 10.1186/s40169-019-0226-9 PMC6441665

[28] van de Veen W , Globinska A , Jansen K , et al. A novel proangiogenic B cell subset is increased in cancer and chronic inflammation. Sci Adv. 2020;6:eaaz3559. 32426497 10.1126/sciadv.aaz3559 PMC7220305

[29] Junttila MR , de Sauvage FJ . Influence of tumour micro‐environment heterogeneity on therapeutic response. Nature. 2013;501:346‐354. 24048067 10.1038/nature12626

[30] Folkman J , Kalluri R . Cancer without disease. Nature. 2004;427:787‐787. 14985739 10.1038/427787a

[31] Kerbel RS . Tumor angiogenesis. N Engl J Med. 2008;358:2039‐2049. 18463380 10.1056/NEJMra0706596 PMC4542009

[32] Finn RS , Qin S , Ikeda M , et al. Atezolizumab plus bevacizumab in unresectable hepatocellular carcinoma. N Engl J Med. 2020;382:1894‐1905. 32402160 10.1056/NEJMoa1915745

[33] Cima I , Kong SL , Sengupta D , et al. Tumor‐derived circulating endothelial cell clusters in colorectal cancer. Sci Transl Med. 2016;8:345ra389. 10.1126/scitranslmed.aad7369 27358499

[34] Wang W , Wang X . A refocus on the advances of single‐cell biomedicine. Cell Biol Toxicol. 2020;36:395‐398. 32779088 10.1007/s10565-020-09551-3 PMC7417105

[35] Wang X . Clinical trans‐omics: an integration of clinical phenomes with molecular multiomics. Cell Biol Toxicol. 2018;34:163‐166. 29691682 10.1007/s10565-018-9431-3

